# Editorial: Exploring HPV’s role in genitourinary cancers beyond cervical cancer

**DOI:** 10.3389/fonc.2025.1658619

**Published:** 2025-07-21

**Authors:** Ana Luísa Teixeira, Francisca Dias, Rui M. Gil da Costa

**Affiliations:** ^1^ Molecular Oncology and Viral Pathology Group, Research Center of Instituto Português de Oncologia (IPO) Porto (CI-IPOP)/RISE@CI-IPOP (Health Research Network), Portuguese Oncology Institute of Porto (IPO Porto), Porto Comprehensive Cancer Center (Porto.CCC), Porto, Portugal; ^2^ Post-Graduate Program in Animal Science, Department of Pathology, State University of Maranhão, São Luís, Brazil; ^3^ Post-Graduate Program in Adult Health, Research Center for Experimental and Clinical Pathophysiology and Pharmacology (NEC), Federal University of Maranhão, São Luís, Brazil; ^4^ Laboratory for Process Engineering, Environment, Biotechnology and Energy (LEPABE), Faculty of Engineering, University of Porto (FEUP), Porto, Portugal; ^5^ Associate Laboratory in Chemical Engineering (ALiCE), FEUP, Porto, Portugal

**Keywords:** prostate cancer, bladder cancer, carcinogenesis, penile cancer, human papillomavirus

The present Research Topic is focused on HPV’s role in genitourinary cancers beyond cervical cancer and aims to explore its potential causal role in lesions not usually associated with this virus. The Research Topic includes one systematic review, four original research articles and 2 case reports.

Aldossary et al. contributed a systematic review article describing the prevalence and genotypical distribution of HPV in women in Saudi Arabia. The authors observed significant geographic variability with the highest prevalence rates found in Riyadh compared with the country’s western regions. The most common genotypes were HPV16 and HPV18, which accounted for 46.3% of infections.

Mumba et al. contributed an original research article exploring the association between smoking habits, HPV and the infiltration of lymphocytes in the stroma of penile carcinomas. The authors reported that HPV-positive tumors showed significantly lower numbers of tumor-infiltrating lymphocytes compared with HPV-negative ones. Interestingly, smokers showed lower numbers of infiltrating CD3+ compared with non-smokers.

Guimarães et al. also presented an original research article on the immunology of penile cancer and reported lower frequencies of T lymphocytes in HPV-positive versus HPV-negative tumors. The authors also reported that HPV-positive tumors were associated with reduced *in vitro* activity of antigen-presenting cells.

Na et al. explored the association between HPV persistence and post-surgical recurrence of cervical lesions. The authors found that persistent HPV infection after surgery was an independent risk factor for postoperative recurrence in early-stage cervical cancer.

Another original research article was contributed by Sleiman Jr. et al., who analyzed the impacts of social determinants on HPV vaccination among economically disadvantaged communities. The authors reported that common vaccine barriers in these communities include lack of information, safety concerns and perceptions of sexual inactivity, and conclude that comprehensive education and intervention to build trust are required to reduce cancer disparities.

Vinokurov et al. reported a case of cervical intraepithelial neoplasia in three members of a single Russian family. Interestingly, the authors reported the presence of alleles associated with increased cervical cancer risk in all three family members. These findings support the role of genetic factors in predisposing specific patients for developing cervical cancer and are expected to contribute to improving the prevention of this diseases.

Zhu et al. contributed a case report of a patient with concurrent cervical intraepithelial neoplasia grade III and urethral cancer, both associated with HPV16 infection. These findings underline the potential complexity of HPV-associated lesions.

Overall, these diverse works contribute to shed light on various aspects of HPV-induced pathology of the genitourinary tract. The diversity of lesions associated with this virus and the biological heterogeneity of each pathological entity poses continued challenges for their clinical management. Preventing HPV-induced cancers also remains challenging, and the works contained in this Research Topic provide valuable clues concerning potential strategies to improve cancer prevention in the coming years.

